# Citation bias in otolaryngology systematic reviews

**DOI:** 10.5195/jmla.2021.736

**Published:** 2021-01-01

**Authors:** Matt Vassar, Austin L. Johnson, Adriana Sharp, Cole Wayant

**Affiliations:** 1 matt.vassar@okstate.edu, Clinical Assistant Professor of Psychiatry and Behavioral Sciences, Department of Psychiatry and Behavioral Sciences, Office of Medical Student Research, Oklahoma State University Center for Health Sciences, Tulsa, OK 74107; 2 austin.johnson14@okstate.edu, Office of Medical Student Research, Oklahoma State University Center for Health Sciences, Tulsa, OK 74107; 3 adriana.sharp@okstate.edu, Office of Medical Student Research, Oklahoma State University Center for Health Sciences, Tulsa, OK 74107; 4 cole.wayant@okstate.edu, Office of Medical Student Research and Department of Biomedical Sciences, Oklahoma State University Center for Health Sciences, Tulsa, OK 74107

## Abstract

**Objective:**

Reproducibility of systemic reviews (SRs) can be hindered by the presence of citation bias. Citation bias may occur when authors of SRs conduct hand-searches of included study reference lists to identify additional studies. Such a practice may lead to exaggerated SR summary effects. The purpose of this paper is to examine the prevalence of hand-searching reference lists in otolaryngology SRs.

**Methods:**

The authors searched for systematic reviews published in eight clinical otolaryngology journals using the Cochrane Library and PubMed, with the date parameter of January 1, 2008, to December 31, 2017. Two independent authors worked separately to extract data from each SR for the following elements: whether reference lists were hand-searched, other kinds of supplemental searching, PRISMA adherence, and funding source. Following extraction, the investigators met to review discrepancies and achieve consensus.

**Results:**

A total of 539 systemic reviews, 502 from clinical journals and 37 from the Cochrane library, were identified. Of those SRs, 72.4% (390/539) hand-searched reference lists, including 97.3% (36/37) of Cochrane reviews. For 228 (58.5%) of the SRs that hand-searched reference lists, no other supplemental search (e.g., search of trial registries) was conducted.

**Conclusions:**

These findings indicate that hand-searching reference lists is a common practice in otolaryngology SRs. Moreover, a majority of studies at risk of citation bias did not attempt to mitigate the bias by conducting additional supplemental searches. The implication is that summary effects in otolaryngology systematic reviews may be biased toward statistically significant findings.

## INTRODUCTION

Systematic reviews (SRs) use comprehensive methodologies to summarize a body of evidence on a clinical topic and, when meta-analysis is appropriate, produce a pooled effect estimate for the included primary studies [1]. Well-conducted SRs are preferentially considered by guideline development panels when weighing evidence for recommendations [2]. While many aspects of the SR process may lead to bias, among the most important steps is the systematic search to locate eligible studies, which can lead to sampling or selection bias if the studies retrieved during the search process do not represent the population of available studies [3]. One particular practice—hand-searching reference lists for additional studies—may locate additional studies outside of the systematic search. However, according to the Cochrane Handbook for Systematic Reviews of Interventions, hand-searching reference lists of included studies may lead to the selective inclusion of statistically significant studies with effect sizes similar to other published studies retrieved from database searching [4]. In plain terms, hand-searching reference lists may result in exaggerated SR effect estimates.

Consider a hypothetical SR in which a comprehensive database search has been conducted. The SR authors may choose to conduct a supplemental search (e.g., a search that complements a database search) to identify additional studies that are relevant to the SR topic. A popular method of supplemental searching is to scan reference lists of studies that are included in the SR [4, 5], despite little evidence to support the practice. Scanning reference lists for potentially relevant studies may increase the number of studies included in the SR but is associated with significant methodological concerns. For one, authors are known to cite studies in an unbalanced manner. One primary motivation for citing studies is to convince readers that one's point of view is correct [6]. Moreover, studies with statistically significant results are more often cited than those with nonsignificant or null findings [7]. Ravnskov reported that trials for lowering cholesterol to prevent coronary heart disease were cited six times more if their results supported lowering cholesterol [8]. Thus, hand-searching references may bias SR summary effects in a unidirectional manner.

Vassar et al. found that supplemental search methods such as a hand-search of medical journals are less biased because they are more likely to retrieve a balanced cohort of studies (e.g., a range of effect sizes and directions), although published literature is likely biased toward positive results and significant effects [9]. However, the Cochrane Handbook recommends hand-searching as a useful adjunct to searching electronic databases because not all trial reports are included in electronic databases or include relevant or easily identifiable search terms in the title or abstracts [10].

To date, there have been few studies examining the extent of hand-searching reference lists in SRs. To address this gap, the authors investigated a broad sample of SRs from one area of medicine—otolaryngology—and quantified the number of SRs that hand-searched references. We also examined whether additional types of supplemental searching that are less biased, such as hand-searching journal issues or trial registries, were conducted. Moreover, we compared the rates of hand-searching reference lists in SRs that mentioned adherence to the Preferred Reporting Items for Systematic Reviews and Meta-Analyses (PRISMA) statement, because PRISMA is associated with higher quality SRs [11]. Last, we investigated whether different funding sources were associated with increased rates of hand-searching reference lists.

## METHODS

We identified SRs and meta-analyses published from January 1, 2008, to December 31, 2017, in the top nine clinical otolaryngology journals based on their H-indexes. This time parameter was chosen to allow an analysis of a ten-year cross-section of SRs, which was deemed sufficient to draw conclusions about the rates of hand-searching. A PubMed search (which includes MEDLINE) was performed by one author using a procedure based on one that was sensitive to identifying SRs and meta-analyses [12] but with modifications to account for recent changes to PubMed indexing. We also included search terms for “meta-regression,” which sometimes appears in titles of SRs and meta-analyses. The journals included in the PubMed search were: *American Journal of Otolaryngology—Head and Neck Medicine and Surgery, Clinical Otolaryngology, Current Opinions in Otolaryngology & Head and Neck Surgery, International Journal of Otolaryngology, JAMA Otolaryngology—Head & Neck Surgery, Journal of the Association for Research in Otolaryngology, Journal of Otolaryngology—Head & Neck Surgery, The Laryngoscope,* and *Otolaryngology—Head and Neck Surgery.* The exact search strategy, used on December 8, 2017, was:

(“JAMA Otolaryngol Head Neck Surg” [Journal] OR “Otolaryngol Head Neck Surg” [Journal] OR “J Assoc Res Otolaryngol” [Journal] OR “Clin Otolaryngol” [Journal] OR “Curr Opin Otolaryngol Head Neck Surg” [Journal] OR “Am J Otolaryngol” [Journal] OR “Int J Otolaryngol” [Journal] OR “J Otolaryngol Head Neck Surg” [Journal] OR “Laryngoscope” [Journal]) AND (metaanalyses [Title/Abstract] OR meta-analysis [Title/Abstract] OR “meta analyses” [Title/Abstract] OR metaanalysis [Title/Abstract] OR “systematic review” [Title] OR meta-regression [Title] OR metaregression [Title] OR meta-analysis [Publication Type]) AND (“2008/01/01” [PDAT] : “2017/12/31” [PDAT])

In 2018, PubMed added a new feature that allowed SRs to be searched as a publication type. This was not included in our search as it predated this update [13]. In addition to our PubMed search, we electronically searched the Cochrane Library using the EBSCOhost platform for Cochrane otolaryngology SRs on December 19, 2017. For this search, we used the same date parameter and filtered our search to only SRs published by the Cochrane Ear Nose and Throat group.

Studies retrieved from the database search were imported to and housed in Rayyan [14], an online article screening platform designed for systematic reviewers. Two authors independently screened all references for inclusion and exclusion while remaining blinded to each other's responses. Discrepancies were resolved by group discussion, and duplicates were removed. Inclusion criteria were SRs published in the journals that we searched. We defined an SR according to the PRISMA-P definition [15].

The following elements were extracted from each SR by two independent authors who maintained blinding to each other's responses: whether reference lists were hand-searched (yes/no), other kinds of supplemental searching (e.g., search of trial registries), mention of adherence to PRISMA guidelines (yes/no), and funding source.

Following extraction, these two authors met to review discrepancies and achieve consensus. Stata 15.1 (STATAcorp) was used to fit a penalized logistic regression model, rather than maximum likelihood, as some predictor variables had low event rates. Prior to analysis, we conducted regression diagnostics, including the variance inflation factor to evaluate for multicollinearity among predictors. All variance inflation factors were in satisfactory ranges and indicated no sign of collinearity. Our regression was designed to investigate the association of adherence to PRISMA (yes/no), Cochrane SR status (yes/no), and funding source (industry, government, private, hospital/university, mixed, none) with hand-searching reference lists. The variables included in the model were chosen to answer whether reporting guidelines and more stringent methodological requirements (i.e., Cochrane and funding source) were associated with rates of hand-searching.

## RESULTS

**Figure 1:**
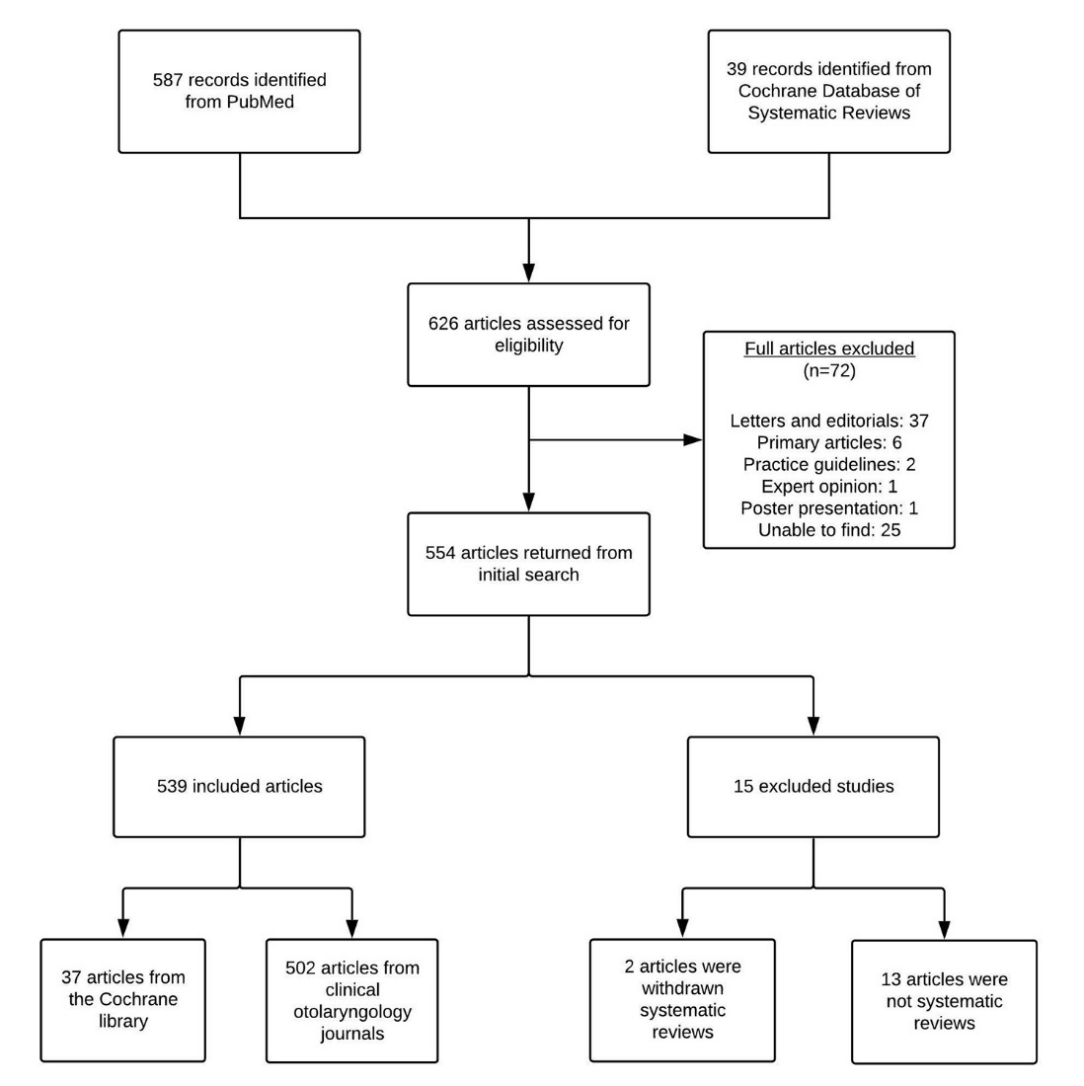
Flow diagram of included and excluded articles

Our search yielded 587 articles from PubMed and 39 articles from the Cochrane Database of Systematic Reviews. Of these 626 articles, 554 were included from our initial screen. A total of 15 were excluded (including 2 duplicates), and 539 were included for analysis: 502 from clinical otolaryngology journals and 37 from the Cochrane library (Figure 1). Of the 539 included SRs, 208 (38.6%) mentioned adherence to PRISMA guidelines. The majority of SRs were either not funded or did not provide a funding disclosure statement (433/539, 80.3%). Of the SRs that mentioned a funding source, the most common source of funding was public entities (e.g., government) (49/106, 46.2%).

Overall, 72.4% (390/539) of SRs hand-searched reference lists, including 97.3% (36/37) of Cochrane reviews. For 228 (58.5%) of the SRs that hand-searched reference lists, no other supplemental search (e.g., search of trial registries) was conducted. There were 162 studies (30.1%) that searched a database, conducted hand-searching, and used other supplementary search methods. No SRs listed the exact articles that were retrieved from a hand-search of reference lists. Logistic regression did not reveal any reliable, statistically significant associations between trial characteristics and the practice of hand-searching reference lists (Table 1).

**Table 1:** Penalized logistic regression of trial characteristics and use of hand-searching reference lists

Characteristic	n	aOR	(95% CI)
Cochrane			
Not Cochrane	502	Reference	
Cochrane	37	10.31	(1.98–53.64)
PRISMA			
No PRISMA adherence	331	Reference	
PRISMA adherence	208	1.02	(0.69–1.51)
Funding source			
None/Not mentioned	433	Reference	
Government	49	0.94	(0.49–1.82)
Private	10	1.41	(0.34–5.91)
Industry	12	0.79	(0.25–2.52)
Hospital/University	11	1.01	(0.29–3.58)
Mixed	24	0.87	(0.36–2.14)

aOR: adjusted odds ratio; CI: confidence interval.

## DISCUSSION

Our results indicate that including studies from reference lists is a methodologically accepted and common practice in otolaryngology SRs, including Cochrane SRs. For the majority of SRs in which reference lists were hand-searched, no other supplemental search was conducted. Many SRs did not specify the articles whose reference lists were searched, which might inhibit the reproducibility of their findings. The implications of these findings were that the summary effects of otolaryngology SRs might be biased toward statistically significant findings. Similar findings exist in the field of dermatology [9].

Hand-searching reference lists is a known source of bias for SRs [4]. This form of bias is easily mitigated by adjusting supplemental search strategies. A previous study looking at complex interventions described the time-intensiveness of SR searching [5]. In that study, the database search took 2 weeks and returned only 35% of the articles included in the final SR sample. Comparing the time invested and the number of articles returned by hand-searching references, by which 41% of the included articles were identified, the authors concluded that database searches might yield fewer results and required significantly more time investment. The authors further stated that hand-searching reference lists was “especially powerful for identifying high quality sources in obscure locations,” which might be true. However, they did not discuss the quality of articles included from hand-searches of reference lists, nor did they discuss the results of the articles that were identified from both database sources and hand-searches of reference lists.

Given the baseline knowledge that studies are cited most often to reinforce a study's findings and that studies with statistically significant findings are more likely to be cited [6, 7], it is possible that these authors could influence future readers to insert citation bias in their SRs, despite that they have used numerous search methods—database, hand-search of journals, hand-search of references, and others—to collect a sample of articles. Thus, we would have preferred to see the authors recommend that readers emulate their methods, because their methods are likely to gather a diverse set of articles, with diverse effect sizes, in multiple directions.

Given our findings, we recommend reevaluation of standard search methods in otolaryngology SRs. The predominant search combination was an electronic database search and a hand-search of included article reference lists. Employing robust search strategies can be time-intensive. Moreover, a Cochrane SR investigating the effectiveness of hand-searching references found that all included studies had a high risk of bias, indicating that no robust data existed to support the practice [16]. Despite that, the Cochrane review authors concluded that hand-searching reference lists might be appropriate in specific circumstances, although these circumstances might be difficult to identify. While the Cochrane handbook mentions the practice of hand-searching references, a Cochrane review questions this practice and instead recommends multiple kinds of supplemental searching [1].

Based on our findings, we encourage systematic reviewers to move away from hand-searching of reference lists due to the potential bias that this creates. However, hand-searching is not necessarily an ineffective method and may be used in concordance with other search methods. Furthermore, we build upon previous work by providing the following recommendations. First, a complete SR search strategy should be established a priori [17]. Second, if supplemental searches are deemed necessary, we recommend authors carefully weigh the benefits and risks of all possible supplemental search methods (e.g., search of trial registries, hand-search of references, hand-search of journals) [18]. Third, we recommend that when authors weigh the pros and cons of supplemental search methods, they adhere to robust guidance, like the Cochrane Handbook, rather than experience and popular or known methods [1]. Last, if a supplemental search is conducted, regardless of its type, we recommend authors disclose which articles were retrieved using these supplemental methods and conduct a sensitivity analysis that removes these articles to quantitatively demonstrate the influence of articles retrieved from a supplemental search on the summary effect [19].
